# Detection of *Escherichia coli* O157:H7 Using Automated Immunomagnetic Separation and Enzyme-Based Colorimetric Assay

**DOI:** 10.3390/s20051395

**Published:** 2020-03-04

**Authors:** Ji Young Park, Kisang Park, Gyeongsik Ok, Hyun-Joo Chang, Tae Jung Park, Sung-Wook Choi, Min-Cheol Lim

**Affiliations:** 1 Department of Chemistry, Chung-Ang University, Seoul 06974, Korea; jrorangece@naver.com (J.Y.P.); tjpark@cau.ac.kr (T.J.P.); 2 Research Group of Consumer Safety, Korea Food Research Institute (KFRI), Jeollabuk-do 55365, Korea; 07938@kfri.re.kr (K.P.); gsok@kfri.re.kr (G.O.); hjchang@kfri.re.kr (H.-J.C.); swchoi@kfri.re.kr (S.-W.C.); 3 Department of Molecular Science and Technology, Ajou University, Gyeonggi-do 16499, Korea

**Keywords:** automation, immunomagnetic separation, colorimetric detection, enzymatic reaction, pathogenic bacteria

## Abstract

The food industry requires rapid and simple detection methods for preventing harm from pathogenic bacteria. Until now, various technologies used to detect foodborne bacteria were time-consuming and laborious. Therefore, we have developed an automated immunomagnetic separation combined with a colorimetric assay for the rapid detection of *E. coli* O157:H7 in food samples. The colorimetric detection method using enzymatic reaction is fascinating because of its simplicity and rapidity and does not need sophisticated devices. Moreover, the proposed procedures for the detection of bacteria in food take less than 3 h including pre-enrichment, separation and detection steps. First, target-specific immunomagnetic beads were introduced to contaminated milk in a pre-enrichment step. Second, the pre-enriched sample solution containing target bacteria bound on immunomagnetic beads was injected into an automated pretreatment system. Subsequently, the immunomagnetic beads along with target bacteria were separated and concentrated into a recovery tube. Finally, released β-galactosidase from *E. coli* O157:H7 after lysis was reacted with chlorophenol red β-galactopyranoside (CPRG) used as a substrate and the colorimetric change of CPRG was determined by absorbance measuring or the naked eye. By the proposed approach in this study, we could detect 3 × 10^2^ CFU/mL of *E. coli* O157:H7 from a milk sample within 3 h.

## 1. Introduction

Enterohemorrhagic *Escherichia coli* O157:H7 (*E. coli* O157:H7) is one of the major foodborne pathogens producing cytotoxins such as verotoxin and shiga toxin. According to the Center for Disease Control and Prevention (CDC), transmission of *E. coli* O157:H7 causing food poisoning is primarily occurred by contaminated food and water. They become a direct cause for increasing financial losses for both the food industry and the consumer [1,2,3]. However, traditional methods for detecting foodborne bacteria can take a few days because these include long enrichment steps for a large sample volume consisting of 25 g of a food sample and 225 mL of a buffer solution, plating to a selective agar and biochemical reaction procedures [4,5,6]. Thus, the methods consist of laborious experimental steps for confirming the bacteria cells and require a trained person to perform the test [7,8]. As a biosensor platform, a nanoporous membrane-based impedimetric sensor was developed for the detection of *E. coli* O157:H7 in milk with a detection limit of 10^2^ CFU/mL [9]. Recently, a nickel oxide thin film based label-free amperometric sensor was reported for the detection of *E. coli* O157:H7 with a detection limit of 10 CFU/mL [10]. However, these electrochemical detection methods require trained personnel and analytical instruments. Therefore, there is a crucial issue for the development of a simple method to provide easy, fast and automatic systems for separating and detecting the foodborne bacteria from food samples [11].

Immunomagnetic separation (IMS) is effective for identifying bacteria from foods due to selective separating target molecules from a heterogeneous matrix [12,13,14,15]. A target-specific antibody on the surface of a magnetic bead is a crucial factor for increasing the yield of selective separation and the concentration of the antigenic target [16,17,18]. Especially, the magnetic beads could be successfully separated and concentrated from the large volume of the sample solution by applying the external magnetic field. Thus, the IMS method offers some advantages in the food safety field including high selectivity and rapidity for detecting a hazardous target by reducing the food matrix effect [19,20,21,22]. Recently, some automated pretreatment devices have been commercialized based on the IMS method. Among them, the Pathatrix^®^ Auto Instrument efficiently concentrates target pathogens from food samples. However, the cultural enrichment step needs more than 5 h for 250 mL of sample volume. Afterward, the finally concentrated sample is inspected by PCR or a selective agar medium. This still needs a long enrichment and complicated analysis procedures [23,24]. The KingFisher Flex Magnetic Particle Processor is also commercialized but the small sample volume is unfit for food safety on the basis of Food Standards [25,26]. And the TECTA™ B16 Automated Microbiology System is designed to detect *E. coli* O157:H7 and total coliforms from water samples. Target bacteria contaminated in waste water at 10 CFU/100 mL could be detected within 24 h by enzyme expression based on signal enhancing [27]. This system is fully automated from enrichment to detection by the activity of enzymes released from the target bacteria. Nonetheless, it is hard to apply on the rapid detection of bacteria in food samples due to the absence of a selective target separation step. Therefore, the food safety field still requires an automated system and applicable method for detecting the foodborne bacteria in food samples.

One of the promising approaches for detecting *E. coli* as microbial pollution is by using a colorant such as chlorophenol red β-galactopyranoside (CPRG) that could be used as substrate by β-galactosidase (β-GAL) of *E. coli.* Specifically, the intracellular β-GAL enzyme breaks down CPRG (yellow color) in to CPR (red magenta color) and galactose. The formation of CPR could be identified by absorbance measuring at 570–595 nm [28,29]. Gunda et al. reported that *E. coli* in water could be detected in the range of 4 × 10^2^–4 × 10^4^ CFU/mL in 1 h by β-GAL based CPRG composition reaction with a micro-filter based bacteria concentration and hydrogel assisted lysis [30]. However, this approach also required an effective pretreatment method for enhancing the performance capacity to a 250 mL sample including 25 g food and 225 mL of buffer solution. Thus, it is also important to eliminate the colorant in food samples to analyze the colorimetric response clearly.

**Figure 1:**
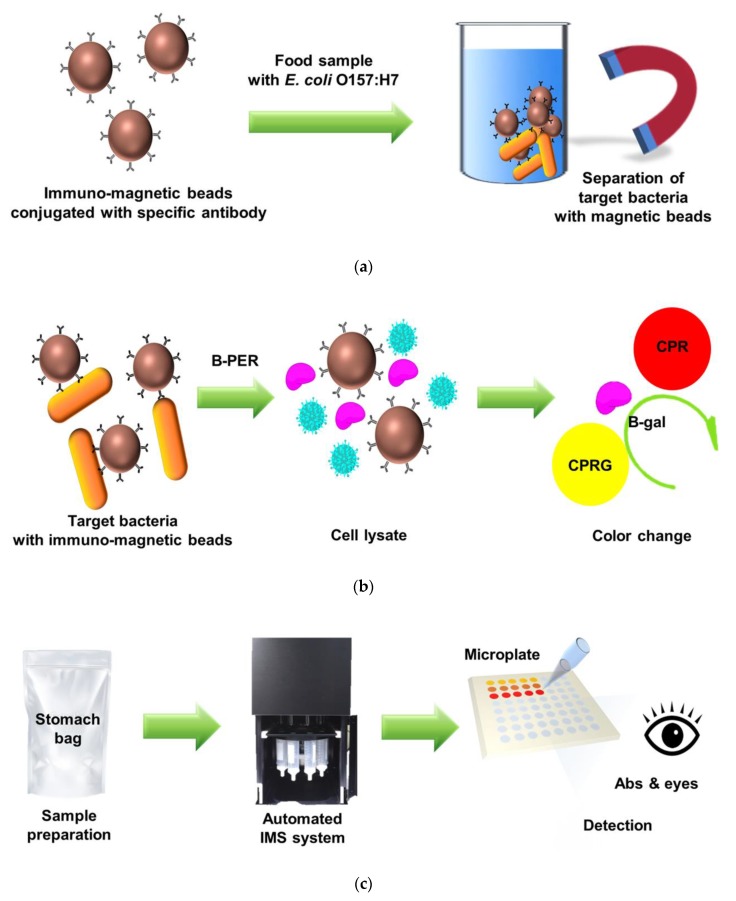
Schematic illustration of experimental procedures: (**a**) The basic principle of the Immunomagnetic separation (IMS) method for separating and concentrating target bacteria from a sample; (**b**) Colorimetric response of β-GAL enzyme-base converting chlorophenol red β-galactopyranoside (CPRG) into CPR after lysis of the target bacteria using a B-PER; (**c**) Proposed detection approach using automated IMS system combined with colorimetric reaction assay. This system is able to detect target *E. coli* O157:H7 via absorbance measuring or the naked eye.

In this study, we present an automated IMS system combined with an enzyme-based colorimetric assay for the rapid detection of pathogenic *E. coli* O157:H7 from food samples in a total 3 h operation process as shown in Figure 1. The target bacteria were effectively separated and concentrated during 30 min of an automated IMS process after 1 h of pre-enrichment and 1 h of target-specific immunomagnetic beads capturing a reaction. Then, passing through the proper lysis of collected bacteria, the yellow-colored CPRG reacted with released β-GAL and changed to red-colored CPR in 30 min. This proposed approach was applied to detected the target *E. coli* O157:H7 form of a 250 mL sample solution at 10^2^ CFU/mL by increasing the number of cellular bacteria in 2 mL of recovery solution volume using the IMS process. The resulting color changes were correlated to the concentration of recovered bacteria from the milk samples and the number of contaminated bacteria could be predicted by absorbance measuring or the naked eye.

## 2. Materials and Methods

### 2.1. Materials

Chlorophenol red β-galactopyranoside (CPRG), Buffered peptone water (BPW), phosphate buffered saline (PBS, pH 7.4) and Tween-20 were purchased from Sigma–Aldrich (St. Louis, MO, USA). B-PER^®^ Bacterial Protein Extraction Reagent (B-PER), Sorbitol MacConkey agar (SMAC), cefixime-tellurite supplement and Falcon tissue cultural treated-96 well microplate were purchased from Thermo Fisher Scientific Inc. (Cambridge, MA, USA). Tryptic soy broth (TSB) for bacteria enrichment was purchased from Becton Dickinson and Company (Franklin Lakes, NJ, USA). For a selective target pathogen separation, a BacTrace anti-*E. coli* O157 magnetic bead was obtained from Kirkegaard and Perry Laboratories (Gaithersburg, MD, USA). Sterile Whirl-Pak bags as food sample containers were purchased from Nasco (Ft. Atkinson, WI, USA). Fresh dairy milk (Namyang Dairy Products Co. Ltd., Seoul, Korea) was purchased from a local supermarket and stored at 4 °C in a refrigerator. The purchased milk as a model food matrix was used before the expiration date.

### 2.2. Bacteria Enrichment Culture and Immunomagnetic Bead Reaction

In this study, *E. coli* O157:H7 (NCTC 12079) was used as a target pathogenic bacterium for the proposed detection approach. A stock solution of *E. coli* O157:H7 was grown in TSB broth for 6 h with gentle shaking and then the same liquid culture was spread on a cefixime-tellurite SMAC plate followed by incubation at 37 °C overnight. A single colony of *E. coli* O157:H7 was picked and incubated in 10 mL of TSB broth at 37 °C for 6 h with gentle shaking. Fresh dairy milk was spiked with prepared bacteria ranging from 10^1^–10^4^ CFU/mL as a final concentration. Then, the artificially contaminated milk sample (25 mL) and BPW broth (75 mL) were placed in a sample bag and incubated for 1 h at 37 °C with constant shaking. To capture and separate the target bacteria, a 500 μL of BacTrace anti-*E. coli* O157 magnetic beads (> 1 × 10^9^ beads/mL) was added to the prepared sample solution and incubated for 1 h at 37 °C with constant shaking.

### 2.3. Immunomagnetic Separation and Concentrationof E. coli O157:H7 in Milk Samples

**Figure 2:**
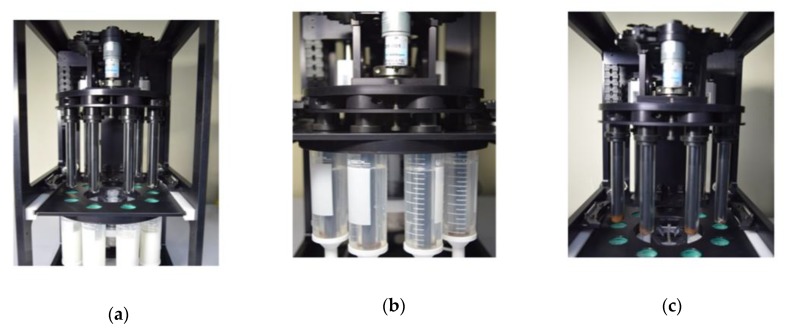

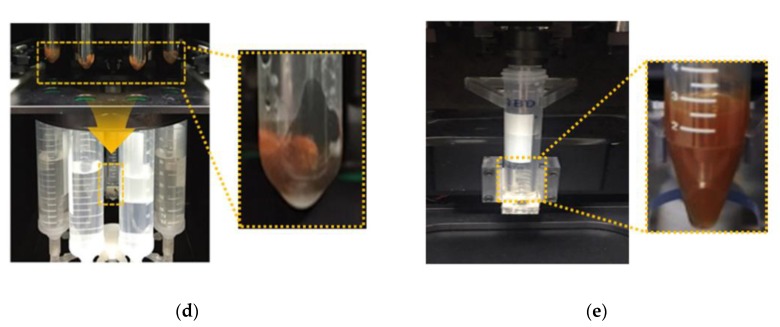
Representative step-by-step images of the automated IMS process: (**a**) Introduction of a pre-enriched sample containing immunomagnetic beads bound with target bacteria in a milk sample; (**b**) Separation of immunomagnetic beads from the sample solution using an inserted magnetic bar; (**c**) Removal of the adhered magnetic beads along with the target bacteria from the sample solution; (**d**,**e**) Immersion of each glass cylinder in to the recovery tube containing 2 mL of buffer solution and redispersion of the magnetic beads and bacteria by vertical movement (two times repetition).

In our previous work, we focused on establishing the basis of an automated IMS system, which validated the examination of an optimum IMS procedure and target bacteria capture efficiency. The performance of the fabricated IMS system was already implemented [11]. The automated IMS system consisted of a fluid control component and a target bacteria recovery component. Figure 2 shows the entire procedure of the bacteria recovery component in the automated IMS system. First, the pre-enriched milk sample (100 mL) with immunomagnetic beads was injected and a 150 mL of BPW containing 0.05% Tween-20 was consecutively flowed using a peristaltic pump. A total 250 mL of sample solution was introduced into eight collection tubes with the same volume in each tube (Figure 2a).

In the next step, the glass cylinder covering the magnetic bar separated the magnetic beads in the injected solution (Figure 2b,c). After the completion of the magnetic beads separation, the immunomagnetic beads were automatically concentrated into a recovery tube containing 2 mL of PBS buffer (Figure 2d,e). The process was repeated twice for collecting the remaining immunomagnetic beads in the sample solution. The target bacteria were spontaneously separated and concentrated in a 30 min of automated IMS process. Thus, the collected target bacteria along with immunomagnetic beads were investigated by field emission scanning electron microscopy (FE-SEM; LEO SUPRA 55, Carl Zeiss, Oberkochen, Germany).

### 2.4. Enzyme-Based Colorimetric Detection of E. coli O157:H7

After the automated IMS process, the 2 mL of recovery solution containing the immunomagnetic beads along with target bacteria was washed twice with PBS buffer (pH7.4) and redispersed in the B-PER lysis solution to release the β-GAL enzyme from the collected bacteria. Then, the appropriate concentration of CPRG solution was added to the bacterial lysate solution and the mixture solution was incubated for 30 min at 37 °C in a dark condition. Finally, the supernatant solution was transferred into a 96-well plate and the absorbance was measured at a 570 nm wavelength by a SpectraMax i3x Multi-mode detection platform from Molecular Devices (Orleans Drive Sunnyvale, CA, USA). To maximize the colorimetric change of the CPRG by the enzymatic β-GAL reaction, some experimental reaction parameters such as CPRG concentration, enzymatic reaction time, sample reaction volume and lysis condition were optimized using the same concentrated *E. coli* O157:H7 culture as the β-GAL source.

## 3. Results and Discussion

### 3.1. Optimization of Enzyme-Based Colorimetric Assay Conditions

To enhance the enzymatic reaction-based color changes, we examined the reaction conditions including the CPRG concentration, B-PER based lysis condition and total reaction volume by measuring the absorbance at a 570 nm wavelength in a 96-well plate. A liquid-based extraction of proteins from cellular bacteria was available to eliminate the use of large equipment such as a probe sonicator and a centrifuge. 

**Figure 3:**
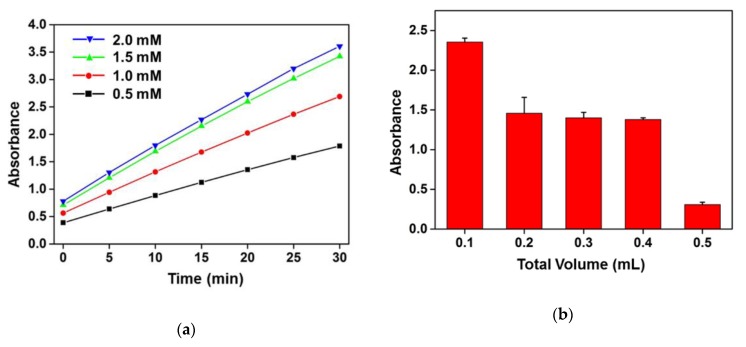
Optimization of enzymatic colorimetric change reactions for the sensitive detection of target bacteria: (**a**) Colorimetric response depending on the CPRG concentration reaction with the same concentrated *E. coli* O157:H7 lysate according to the duration of reaction time (up to 30 min); (**b**) Absorbance responses upon the same amount of CPRG and *E. coli* O157:H7 lysate in various total reaction volumes from 0.1–0.5 mL after 30 min of reaction time.

In this study, B-PER as a lysis solution saved the process time for extracting the β-GAL enzymes from *E. coli* cells because it took a few minutes to lyse the bacterial cell wall. The released protein in the solution was also examined by Bradford Assay after spinning down the cell debris (data not shown here). Thus, the mild lysis condition was also advantageous to enhance the total β-GAL enzyme activity for the efficient conversion of the CPRG into CPR in the same reaction time. The concentration of CPRG as a colorimetric reaction substrate was an important factor influencing the degree of absorbance changes. As shown in Figure 3a, the absorbance increased with a higher concentration of the CPRG and a longer duration of enzymatic reaction. All of the experiments were conducted with the same concentrated *E. coli* O157:H7 culture as β-GAL enzyme source in B-PER lysis solution. The absorbance of 0.5 mM (black line) and 1 mM (red line) of CPRG after enzymatic reaction was lower than that of 1.5 mM (green line) and 2 mM (blue line) of the CPRG concentration. The absorbance tended to saturate over the 1.5 mM of CPRG concentration in the same reaction condition and duration time. Thus, we chose the 1.5 mM of CRPG concentration as the colorant source in the enzyme reaction condition. The enzyme-based colorimetric change reaction was conducted in 0.1–0.5 mL of total volume under the same amount of *E. coli* O157:H7 and CPRG to find the optimum volume reaction solution. As shown in the Figure 3b, the smaller volume of the total reaction solution was more suitable to identify the existence of the target bacteria by observing the colorimetric changes and 0.1 mL of B-PER solution was chosen for the optimum reaction volume.

**Figure 4:**
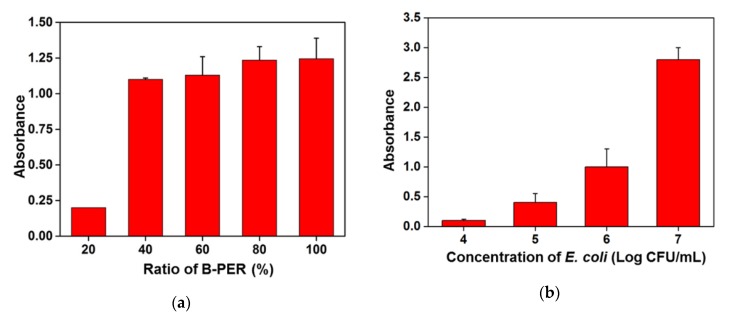
Optimization of enzymatic colorimetric change reactions for the sensitive detection of target bacteria: (**a**) Colorimetric response depending on the mixing ratio between B-PER and 5 mM PBS for the lysis of target bacteria; (**b**) Comparison of absorbance in the range 10^4^ to 10^7^ CFU/mL of target bacteria under the optimized experimental conditions.

To check the lysis ability and effect on the absorbance changes, the proportion of B-PER in lysis solution was tested using a 5 mM PBS as diluent. As shown in the Figure 4a, a 20%–100% of B-PER solution was prepared with a 5 mM PBS and tested to find the optimum concentration of B-PER for effective lysis of the bacteria. When 20% of B-PER was used as a lysis buffer solution, the absorbance slightly increased after the enzyme-based substrate converting reaction finished. There was a small difference in the absorbance changes after enzymatic reaction for the 40% to 100% of B-PER condition. Thus, the 80% of B-PER solution was chosen for the lysis condition to extract the β-GAL enzyme from *E. coli* O157:H7. In accordance with the optimum conditions mentioned above, the absorbance depending on the conversion of CPRG into CPR in various concentrations of *E. coli* O157:H7 was measured after finishing the enzymatic reaction for 30 min. As shown in Figure 4b, the absorbance of reaction solution containing 10^4^ CFU/mL of bacteria was slightly increased. However, the absorbance of CPR dramatically increased from 10^5^ CFU/mL in the bacteria concentration-depending manner. These results indicated that the concentration of target bacteria at the lysis step should be higher than 10^5^ CFU/mL to identify the presence of *E. coli* O157:H7 by the enzyme reaction-based colorimetric changes.

### 3.2. Colorimetric Detection of E. coli O157:H7 in Milk Using the Automated IMS System 

**Figure 5:**
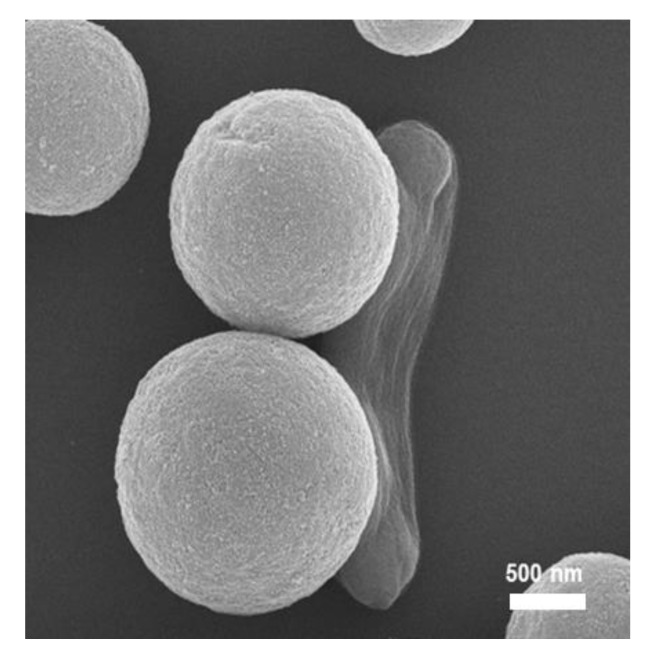
Representative SEM image of target *E. coli* O157:H7 captured by immunomagnetic beads from milk samples after the automated IMS process (Scale bar is 500 nm).

The efficiency of the colorimetric detection of *E. coli* O157:H7 in milk samples was tested using the automated IMS system to separate and concentrate the target bacteria. The performance of the developed IMS system was investigated with each process and optimized in our previous study [11]. The whole milk sample (25 mL) was spiked with a known number of *E. coli* O157:H7 and mixed with BPW (75 mL, containing 0.05% Tween-20). Pre-enriched milk containing immunomagnetic beads was introduced to the automated IMS system with an additional 150 mL of BPW containing 0.05% Tween-20. After finishing the separation and concentration process in 30 min, the collected immunomagnetic beads with target bacteria were redispersed in B-PER solution to release the proteins from the cellular bacteria. Thus, the collected immunomagnetic beads along with the target bacteria were investigated by SEM as shown in Figure 5. Representative SEM images showed the attached target bacteria on the surface of immunomagnetic beads. The results indicated that the immunomagnetic separation based automatic pretreatment system could collect the target bacteria from a large volume of the food sample. The selective medium agar plate based colony counting was carried out to confirm the detection results of the enzyme-based colorimetric assay.

**Figure 6:**
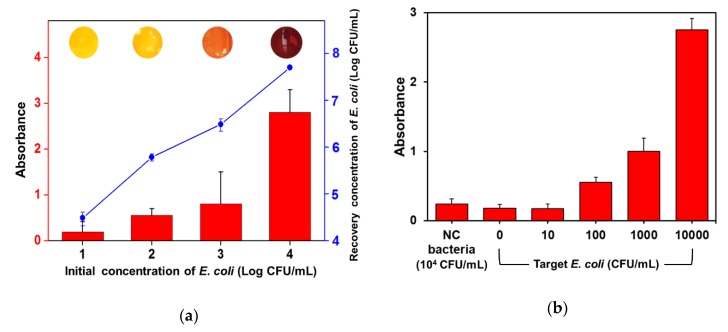
Enzyme reaction based colorimetric detection of *E. coli* O157:H7 from milk samples: (**a**) Recovery concentration of bacteria evaluated by colony counting using a selective agar medium (blue line) was compared with the absorbance of the assay solution (red bar) and final color response (inset digital images) by enzymatic reaction; (**b**) Target bacteria concentration dependent increase of absorbance was evident for β-GAL producing *E. coli* O157:H7, while the absorbance value for the negative control (NC) bacteria (*S. aureus*) tested at 10^4^ CFU/mL was negligible.

The capability of the automated IMS system to separate and concentrate the target bacteria in milk is shown in Figure 6a as the recovery concentration of target bacteria from the colony counting assay. The concentration of finally collected bacteria in 2 mL of solution after the automated IMS process with 2 h of pre-enrichment was effectively increased compared with the initial concentration of target bacteria. These results mean that the recovery yield of the target bacteria using the developed IMS system and constructed method was more than 1000-fold compared with the initial concentration of target bacteria in the range of 10^1^–10^4^ CFU/mL. The recovered concentration of bacteria at 10^2^ CFU/mL of the initial concentration was more than 10^5^ CFU/mL as a critical concentration to discriminate the enzyme reaction based colorimetric changes shown in Figure 4b. Thus, the effect of biologically-relevant analytes on the absorbance monitoring at 570 nm wavelength was investigated by using *Staphylococcus aureus* (*S. aureus*) as negative control bacteria and a fresh milk sample without artificial contamination (Figure 6b). Although the *S. aureus* was tested at 10^4^ CFU/mL in milk with the presence of CPRG, the effect of released biomolecules by cell lysis was negligible. The absorbance for the non-target bacteria containing sample was comparable with that of the fresh milk sample (0 CFU/mL). The target *E. coli* O157:H7 was detected by the enzyme-based colorimetric assay from 3 × 10^2^ CFU/mL of the initial concentration in milk with a high reproducibility.

Colorimetric detection methods have advantages not only in quantitative analysis such as absorbance measuring but also in the discrimination of the final detection results by the naked eye without additional equipment [31,32,33]. In this study, the obtained assay results could also be distinguished by the naked eye as a corresponded color of the inset digital images in Figure 6a. In comparison with the absorbance data, the color was discriminable as light orange (10^2^ CFU/mL), orange (10^3^ CFU/mL) and red (10^4^ CFU/mL), respectively. The color changes induced by the released enzymes from low numbered bacteria were not able to be detected in 30 min. However, the color change of the CPRG solution could clearly be distinguished from 10^3^ CFU/mL of the initial target bacteria concentration by the naked eye. Thus, the low numbered target bacteria might be visually detected with an increased cellular number by a pre-enrichment of more than 2 h. 

To confirm the specific colorimetric response on the target bacteria, the detection assay described above was evaluated using non-target bacteria such as *Salmonella enterica* (*S. enterica*) and *Staphylococcus aureus* (*S. aureus*). Table 1 shows the obtained results on the target-specific colorimetric response after performing the automated IMS process and enzyme reaction using 10^4^ CFU/mL of initial concentration for each tested bacteria strain in milk samples. Critical yellow to red color changes were induced on the samples containing the target *E. coli* O157:H7 while no color changes occurred on the negative control samples containing only non-target bacteria such as *S. enterica* and *S. aureus*. These results indicated that the non-target bacteria did not interrupt the specific enzymatic reaction to identify the presence of target *E. coli* O157:H7 in the food samples.

## 4. Conclusions

In this study, we proposed a rapid and easy detection method for *E. coli* O157:H7 using an automated IMS system and enzyme-based colorimetric assay. The advantage of the combined process is the detection of target bacteria in a quantitative manner through numerical values from absorbance measuring and in a qualitative manner by color information seen by the naked eye. Thus, this process could concentrate the target bacteria from food samples and enhance the specific colorimetric response at the same working time, which does not require sophisticated and long analysis procedures such as traditional culture-based analysis. Moreover, the target bacteria in food samples was effectively separated and concentrated into confined recovery buffer solutions by the automated IMS process using target-specific immunomagnetic beads. Significant numbers of target bacteria were also confirmed by the colorimetric response using a specific enzyme reaction within 30 min after the pre-enrichment and the automated IMS process. We demonstrated that the proposed procedure could detect target *E. coli* O157:H7 at the initial concentration of 3 × 10^2^ CFU/mL in milk samples with a specific response. As a result, we anticipate that the designed process will be a promising on-site detection method such as paper-based strip sensors to identify the pathogenic bacteria in foods.

## Figures and Tables

**Table 1:** Specific colorimetric response on target *E. coli* O157:H7 after immunomagnetic separation and enzymatic reaction. *Salmonella enterica* (*S. enterica*) and *Staphylococcus aureus* (*S. aureus*) were used as non-target bacteria strains (10^4^ CFU/mL in milk samples).

Bacterial Strains	Colorimetric Change	Absorbance Change	Final Reaction Solution Color
*E. coli O157:H7*	Yes	Yes	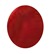
*S. enterica*	No	No	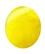
*S. aureus*	No	No	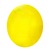
*E. coli O157:H7* + *S. enterica*	Yes	Yes	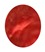
*E. coli O157:H7* + *S. aureus*	Yes	Yes	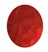
